# Tait’s Operation

**Published:** 1883-03

**Authors:** T. Gaillard Thomas

**Affiliations:** New York; Professor of Gynæcology in the College of Physicians and Surgeons, New York


### TAIT'S OPERATION.

### A Contribution to the Subject of the Removal of the Uterine Appendages (Tait's Operation) for Prolonged Menstrual Troubles, with Recurrent Pelvic Inflammations.*

T. Gaillard Thomas, M. D., New York.

*Professor of Gynæcology in the College of Physicians and Surgeons, New York.*

In the issue of the *British Medical Journal* for July 29th, 1882, appeared a remarkable essay by Mr. Lawson Tait, of Birmingham, England, enunciating views entirely at variance with those heretofore held by the medical profession, advocating pathological tenets which were altogether new, and recommending a plan of treatment which is at once original and bold.

This article was entitled " Remarks on the Diagnosis and Treatment of Chronic Inflammation of the Ovary," and I refer to it at some length to-night, first, because my own paper is based upon its suggestions; second, because I feel sure that the positions which it assumes will interest the Fellows of the Academy, even if they deny their validity; and, third, because I myself feel convinced that this bold and original surgeon has advanced views which are destined to open a new field for gynæcological surgery in the future and to exert upon this department of medicine an enduring influence.

I propose to give to-night, in as succinct a manner as possible, those points in the paper which are original with Mr. Tait; and I would say here that, while I do not by any means feel warranted by my own experience and observation in accepting all of them, I do believe that there is a sufficient amount of truth in some of them to make the essay one of the most valuable which this decade has produced for the gynæcologist.

Since that period which constituted so remarkable an era in the history of gynæcology—when Henry Bennet, of England; Simpson, of Scotland; and Marion-Sims, of America, brought their eminent labors to bear upon this department of medicine—until the present

*Read, with the exhibition of specimens, before the New York Academy of Medicine, December 21st, 1882. We quote these remarks of Dr. Thomas, as they give briefly the new pathological notions of Dr. Tait, which are interesting, and because it shows to what butchery allopathy is driven *to cure* its patients.—Ed. H. P.

time, a vast deal of attention has been directed to the uterus, the vagina, the uterine ligaments, and the pelvic peritonæum and areolar tissue; while diseases of the ovaries and Fallopian tubes have been left enveloped by a cloud of ignorance and uncertainty which few have endeavored to penetrate and dispel. Tilt, of London, the firm, persistent, and able advocate of the claims of ovarian pathology, has during this time stood almost alone, steadily and consistently enunciating views with which few sympathized and still fewer indorsed and sustained. At the present time it may safely be stated that a wholesome revolution is occurring in this respect, that reflective men devoted to gynæcology are now recognizing that a very large proportion of cases of menstrual disorder, pelvic neuralgia, and diffi ·ult locomotion, which were formerly attributed to the uterus, its ligaments, or the pelvic areolar tissue, are entirely due to ovarian disorder, and that hundreds of cases in which that devoted organ, the uterus, was cut, cauterized, and leeched had the same pathological origin. Mr. Tait's paper deals with this most prolific subject, and for this reason, added to those which I have mentioned, must be regarded as a most opportune effort to cast light where the darkness is most dense in gynæcology, and to offer aid in a class of cases in which the operator in this department needs most assistance.

The following points may be given as the most original and valuable of the views enunciated by Mr. Tait:

1. He assumes that the view formerly held—that laparotomy operations should be postponed until absolute risk to the life of the patient rendered them necessary—should now be abandoned; and that, in the hands of an expert, they are at present so free from danger as to be justifiable even when life is not jeopardized.

2. That the usually accepted doctrine of the dependence of menstruation upon ovulation is wholly erroneous.

3. That the ovaries have nothing whatever to do with menstruation, and that this phenomenon is dependent upon the Fallopian tubes.

4. That many cases of abnormal menstruation are justifiably treated and are relievable in no other way than by extirpation of the ovaries and tubes.

5. That in chronic ovarian disease the tubes are invariably involved, and that in most cases it is the tubes which are chiefly at fault.

6. That the mortality in his last thirty-five operations having been only one, even this slight loss of life is susceptible of diminution in the future.

7. That many of those cases heretofore regarded as instances of menstrual, or recurrent, pelvic peritonitis or cellulitis are really due to tubal dropsy and ovarian disease, an occasional discharge of the purulent accumulation of the former giving rise to slight and passing attacks of these affections.

This last statement Mr. Tait does not make in the paper to which I have alluded. I am indebted for it to my friend Dr. Emmet, who, in a recent visit to Europe, obtained it from a personal conversation with that gentleman.

